# Development of a Nomogram for Predicting the Cumulative Incidence of Disease Recurrence of AML After Allo-HSCT

**DOI:** 10.3389/fonc.2021.732088

**Published:** 2021-09-27

**Authors:** Tongtong Zhang, Xiebing Bao, Huiying Qiu, Xiaowen Tang, Yue Han, Chengcheng Fu, Aining Sun, Changgeng Ruan, Depei Wu, Suning Chen, Yang Xu

**Affiliations:** ^1^ Jiangsu Institute of Haematology, Key Laboratory of Thrombosis and Haemostasis of Ministry of Health, The First Affiliated Hospital of Soochow University, Suzhou, China; ^2^ National Clinical Research Centre for Haematological Diseases, Suzhou, China; ^3^ Institute of Blood and Marrow Transplantation, Collaborative Innovation Centre of Haematology, Soochow University, Suzhou, China

**Keywords:** allogeneic hematopoietic cell transplant, acute myeloid leukemia, predictive mutations, gene mutation topography, cumulative incidence of relapse

## Abstract

Using targeted exome sequencing, we studied correlations between mutations at diagnosis and transplant outcomes in 332 subjects with acute myeloid leukemia (AML) receiving allotransplantation. A total of 299 patients (299/332, 90.1%) had at least one oncogenic point mutation. In multivariable analyses, pretransplant disease status, minimal residual disease (MRD) before transplantation (pre-MRD), cytogenetic risk classification, and *TP53* and *FLT3-ITD* ^high ratio^ mutations were independent risk factors for AML recurrence after allotransplantation (p < 0.05). A nomogram for the cumulative incidence of relapse (CIR) that integrated all the predictors in the multivariable model was then constructed, and the concordance index (C-index) values at 6, 12, 18, and 24 months for CIR prediction were 0.754, 0.730, 0.715, and 0.690, respectively. Moreover, calibration plots showed good agreements between the actual observation and the nomogram prediction for the 6, 12, 18, and 24 months posttransplantation CIR in the internal validation. The integrated calibration index (ICI) values were 0.008, 0.055, 0.094, and 0.136 at 6, 12, 18, and 24 months posttransplantation, respectively. With a median cutoff score of 9.73 from the nomogram, all patients could be divided into two groups, and the differences in 2-year CIR, disease-free survival (DFS), and overall survival (OS) between these two groups were significant (p < 0.05). Taken together, the results of our study indicate that gene mutations could help to predict the outcomes of patients with AML receiving allotransplantation.

## Introduction

Acute myeloid leukemia (AML) is an aggressive disorder with heterogeneous morphology and genetic aberrations detected in leukemic cells (1). The outcome of AML patients has improved significantly over the last three decades because of improvements in clinical management, such as supportive care, targeted therapy, and the increasing use of allogeneic hematopoietic stem cell transplantation (allo-HSCT) (2–4). Allo-HSCT is a curative therapeutic option for patients with AML, especially for those with intermediate or high risk. Although allo-HSCT shows a strong graft-*versus*-leukemia (GVL) effect in AML patients, the overall survival (OS) can still be compromised by disease relapse and/or treatment-related mortality (TRM) (5). Hence, a systemic or comprehensive evaluation of AML patients who underwent allo-HSCT is essential to improve clinical outcomes. Based on accumulating evidence, patients who have not achieved remission status at the time of transplant have a higher relapse rate, suggesting that disease status could drive the choice of conditioning regimen (6). Similarly, patients with poor-risk cytogenetics are known to fare worse after allo-HSCT than patients with a more favorable karyotype (7). Other prognostic factors previously identified include the age of onset and time interval from diagnosis to transplant (6, 8). Despite dramatic advances in the understanding of AML, including significant improvements in prognostic classification, conditioning chemotherapy, and supportive care following allo-HSCT, leukemia relapse remains a significant and daunting clinical problem, and accurately predicting the maximum benefit of allo-HSCT remains a challenge.

In recent years, molecular profiling using next-generation sequencing (NGS)-based myeloid panels targeting recurrent mutations, including but not limited to *NPM1*, *FLT3-ITD*, biallelic mutated *CEBPA*, *ASXL1*, *RUNX1*, *IDH1/2*, and *TP53*, has been performed. The findings from these studies had a significant impact on AML patients’ risk stratification and clinical management (9–13). Although some of these somatic mutations, i.e., mutations in *NPM1*, *FLT3*, and *DNMT3A*, have been taken into consideration in predicting disease relapse in AML patients after allo-HSCT (14–17), a comprehensive prediction model that systemically integrates recurrent mutational profiling for disease relapse is needed in AML with allo-HSCT. In this study, we performed targeted-capture sequencing on *de novo* bone marrow (BM) DNA samples from a large cohort of AML patients to investigate the correlation between mutation topography and the outcome of allotransplants for AML.

## Patients and Methods

### Patients and Study Design

From May 2010 to July 2018, a total of 332 AML patients who received allo-HSCT at our center were involved in this study. A total of 274 (82.5%) patients received induction chemotherapy consisting of standard first-line treatment with an IA (idarubicin and cytarabine)-like regimen composed of 8–12 mg/m^2^ idarubicin (days 1–3) and 100 mg/m^2^ cytarabine (days 1–7). After achieving first complete remission (CR1), patients received consolidation of either at least four cycles of intermediate/high-dose cytarabine-based combination chemotherapy (1–2 g/m^2^ for 3 days) or allo-HSCT treatment. Twenty-one (6.3%) patients underwent allo-HSCT after achieving the second/third/fourth CR (CR2/CR3/CR4), and the remaining 37 (11.1%) patients underwent salvage HSCT when they relapsed or failed to achieve CR. All patients (n = 332) received myeloablative conditioning (MAC) regimens consisting of cytarabine (2 g/m^2^/day for 2 days), busulfan (3.2 mg/kg/day for 3 days), and cyclophosphamide (1.8 g/m^2^/day for 2 days). Patients received a median of 8.15 × 10^8^/kg mononuclear cells (MNCs) (range 1.28–28.52) and a median of 3.49 × 10^6^/kg CD34 cells (range 1.21–10.31). Graft-*versus*-host disease (GVHD) prophylaxis consisted of cyclosporine (CSA) and methotrexate (MTX). In addition, anti-thymocyte globulin (ATG) and mycophenolate mofetil (MMF) were administered to patients undergoing human leukocyte antigen (HLA) mismatched transplant.

These consecutively admitted patients all participated in clinical trials (CHiCTR-OCH-14004612), and their BM and peripheral blood samples were obtained at the time of diagnosis for molecular characteristics by NGS. After exclusions, 320 of 332 patients with the MA regimen were included in the last predicted nomogram (**Supplementary Figure S1**), and the internal validation of random resampling was performed in the meantime.

### Variables Included in the Analyses

The data analysis included patient-related variables, such as age, sex (male *vs*. female), pretransplant disease stage [CR1 *vs.* >CR1 vs. non-remission (NR)], cytogenetic classification (favorable *vs*. intermediate *vs*. adverse), Karnofsky Performance Scale (KPS) score (≥90 *vs*. <90), and minimal residual disease before transplantation (pre-MRD) (positive *vs*. negative). Transplant-related variables included donor type, gender type-matched of donor, ABO blood type-matched, and time from diagnosis to transplant in months. Moreover, the characteristics of individual gene mutations identified with NGS were also included in the analyses.

Using the refined Disease Risk Index (DRI) (18) and European LeukemiaNet (ELN) risk recommendations, 332 patients were classified accordingly into different risk groups. For the cytogenetic classification, we referred to the ELN-2017 and the revised Medical Research Council (MRC) prognostic classification (19). Relapse was defined as the recurrence of >5% BM blasts, the reappearance of blasts in the blood, or the development of extramedullary disease. In addition, the *FLT3-ITD* allelic ratio was determined as the ratio of the *FLT3-ITD* mutant divided by the FLT3 wild type.

### Pre-MRD Monitoring and Definition

Pre-MRD was measured using multiparameter flow cytometry (MFC) and polymerase chain reaction (PCR) within 1 month before transplantation. Almost all patients (except for 12 patients) underwent an MRD test involving leukemia-associated immunophenotypic patterns (LAIPs) using eight color MFCs before allo-HSCT, and the median value of all detected patients (10^-3^) was defined as the cutoff value. For those with leukemic fusion genes (e.g., *RUNX1-RUNX1T1*, *CBFB-MYH11*, *MLL* gene fusions), we defined the PCR-based MRD as a priority and the non-detectable transcripts of these fusion genes as pre-MRD negative. Ninety-eight patients were evaluated MRD by quantitative PCR of special fusion gene transcripts, 10 of them with MFC-MRD negative but detected fusion gene transcripts were defined as pre-MRD positive. Besides, we also considered the Wilms tumor 1 (*WT-1*) as an alternative target for PCR-based MRD, and a value of 200 copies/10^4^ copies of *ABL* was set as the cutoff value. Among 222 patients without performing the special fusion gene transcripts, pre-MRD monitoring evaluated by MFC-MRD and *WT-1* expression. There were 15 patients with MFC-MRD negative but *WT-1* expression positive, who were also defined as pre-MRD positive (details given in **Supplementary Table S1**). Twelve patients did not receive any of the three tests within 1 month before transplantation and were eliminated from the final nomogram.

### Sample Processing, DNA Sequencing, and Mutation Analysis

Targeted sequencing of the entire coding sequences of 382 known or putative mutational gene targets in hematological malignancies (**Supplementary Table S2**) was performed on samples collected from 225 patients at diagnosis and accomplished by a commercial company. Briefly, the genomic DNA was sheared, and the sample libraries were prepared using the TruSeq DNA Sample Preparation Kit (Illumina, San Diego, CA). The libraries were sequenced as paired 150-bp reads on an Illumina HiSeq 4000 according to the manufacturer’s instructions. The average depth of DNA sequencing was 600×. Detected variants were subjected to a rigorous manual curation process, including querying variant databases [e.g., SNP database (dbSNP), the Exome Aggregation Consortium (ExAC), Catalogue Of Somatic Mutations In Cancer (COSMIC), 1000 Genomes Project] and literature reviews. Synonymous variants, variants located outside protein-coding regions, and variants with an allelic fraction (VAF) lower than 1% were filtered.

**Figure 1:**
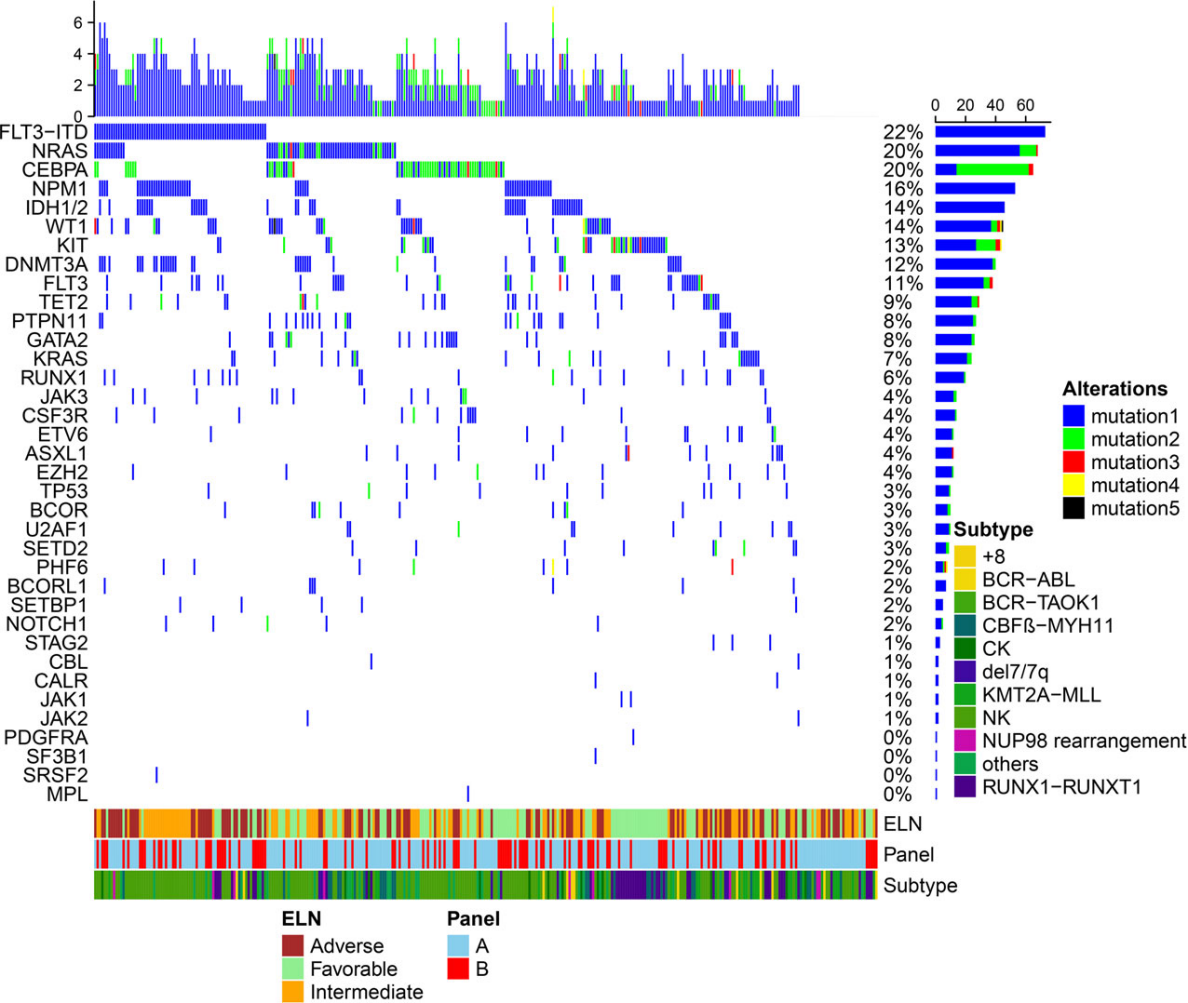
Mutation patterns observed in 332 acute myeloid leukemia patients who were treated with allogeneic hematopoietic stem cell transplantation. **(A)** indicates that 225 samples were performed using the Illumina HiSeq Sequencing platform in Nanjing Shihe Jiyin Biotech Inc. Laboratory. **(B)** indicates that 107 samples were performed by the Ion S5 system (Personal Genome Machine, Thermo Fisher, Grand Island, NY, USA) in our own experimental platform. The plot represents a graphical summary of the distribution of somatic lesions in sequenced genes across the set of patients. Columns represent samples, and rows represent genes. European LeukemiaNet (ELN) risk classification and subtype of chromosome were summarized on the bottom. (The eight mutations consisting of *BIRC3*, *BRAF*, *CDKN2A*, *IL7R*, *MYD88*, *PAX5*, *PDGFRB*, and *PTEN* were not detected in both groups of samples and were not included in the curve).

In other patients (n = 107), targeted ultradeep sequencing of 49 recurrent mutated AML genes was performed by the Ion S5 system (Personal Genome Machine, Thermo Fisher, Grand Island, NY, USA) using our own experimental platform. All 107 samples were also collected at diagnosis. Libraries were prepared using Ion AmpliSeqTM Library Kits 2.0 to obtain 200-bp fragments flanked by adapter and barcode sequences, allowing sequencing and sample identification, respectively. Furthermore, sequencing was performed with Ion PGM in a 200-bp configuration run using a 540 chip. A satisfactory depth of coverage was obtained for all exons (average: 736–4,280, mean: 2,000). Verification of the sequencing results was performed by direct Sanger sequencing. *NPM1* and *CEBPA* mutations were determined by PCR amplification followed by direct bidirectional DNA sequencing. Considering the differences between the two sequencing methods used for the assessment of recurrent mutated genes, we finally selected 45 common genes for statistical analysis. (Note: **Figure 1** shows only 37 of the mutated genes.)

### Statistical Analysis

All patients were followed up from the date of transplant to the date of last examination or death until December 2019. The primary endpoint for this study was cumulative incidence of relapse (CIR), which was calculated from the date of transplant to the first leukemia recurrence, with events of non-relapse mortality (NRM) after transplant being the competing risk. The second endpoints included NRM, disease-free survival (DFS), and overall survival (OS). Events of NRM were defined as death without evidence of leukemia recurrence after transplant. The cumulative incidence function (CIF) was used to show the probability of CIR or NRM, and the differences between the groups were estimated by Gray’s test. The restricted cubic spline was used to flexibly fit the model and visualize the relation of continuous variables with the CIR. A random survival forest (RSF) for leukemia recurrence was performed to screen the candidate predictors first by two indicators: minimal depth (MD) and variable importance (VIMP). The smaller the MD value of a factor is, the greater its prediction ability. VIMP is a comparable measurement of a factor in predicting the response or causal effect and is decreased with the increase in prediction error if the factor is randomized. The method of the RSF model is described in detail in the **Supplementary Data**. Then, these candidate predictors were used to formulate the predictive nomogram for leukemia recurrence after transplant by backward elimination with the Akaike information criterion (AIC) for selecting predictors in the last model. Values of variance inflation factor (VIF) were used to evaluate multicollinearity between variables, with VIF >10 considered indicative of multicollinearity. The interaction and subgroup analyses were also tested and confirmed in the predictive model. Values and plot of the time-dependent concordance index (C-index) were used to measure the discrimination performance of the predicted nomogram. Three numeric metrics of calibration were reported to detect the agreement between the predicted and actual probabilities. The integrated calibration index (ICI) is equivalent to the mean difference between the predicted and actual probabilities (20). E50 and E90 indicate the median and 90th percentile of the difference between the probabilities of observation and prediction. Calibration curves were also drawn by comparing nomogram-predicted probabilities with the observed probabilities of CIR in the internal validation using a bootstrapping method with 1,000 resamples for accurate validation. Decision Curve Analysis (DCA) (21) and net benefits were performed at different thresholds to measure the discrimination and clinical usefulness of the predicted models. The curves of DCA were also plotted for the risk models classified by refined DRI and ELN-2017 recommendations.

Statistical analyses were performed with R version 3.6.0 software (The R Foundation for Statistical Computing, Vienna, Austria; www.rproject.org) *via* the randomForestSRC package for screening and choosing variables and finding interactions between pairs of variables, the packages cmprsk, mstate, and rms packages for establishing the model and nomogram, the pec package for examining the applicability of the model, and the survival package for analysing DFS and OS. A p-value less than 0.05 was considered to be significant.

## Results

### Patients’ Characteristics and Study Design

The characteristics of the 332 patients with AML are summarized in **Table 1**. The median age for the entire group was 35 years (range 12–60 years), and there were 202 males and 130 females. The majority of the patients had a KPS score of 90 or higher (n = 293, 88.3%). Nearly half of the patients (n = 153, 46.1%) had a normal karyotype, and 23.2% had core binding factor leukemia (CBF-AML, n = 77). According to the cytogenetic risk classification: favorable, intermediate, and adverse categories comprised 77 (23.2%), 198 (59.6%), and 57 (17.2%) patients, respectively. At a median follow-up of 30 months (range 0.5–67.0 months), 81 patients (81/332, 24.4%) developed disease recurrence. Relapse occurred at a median of 7.0 months after transplantation, and the interval from transplantation to relapse was ≤6 months for 36 patients (36/81, 44.4%), 6–12 months for 17 (17/81, 21.0%), 12–24 months for 22 (22/81, 27.2%), and ≥24 months for six (6/81, 7.4%). Moreover, the probabilities for 2-year OS, DFS, CIR, and NRM in all patients were 72.1%, 62.6%, 24.2%, and 13.2%, respectively.

**Table 1:** Demographic and clinicopathologic characteristics of the 332 AML patients undergoing allogeneic HSCT.

Demographic or Characteristic	No. of patients	%
Gender		
Male	202	60.8
Female	130	39.2
Age		
<50	300	90.4
≥50	32	9.6
WBC (*10E9/L)		
<10	95	28.6
10–30	82	24.7
≥30	145	43.7
Missing	10	3.0
Cytogenetics risk classification		
Favorable	77	23.2
Intermediate	198	59.6
Adverse	57	17.2
Pre-MRD		
MRDpos	98	29.5
MRDneg	222	66.9
missing	12^*^	3.6
Stage at HSCT		
CR1	274	82.5
>CR1	58	17.5
KPS		
≥90	293	88.3
<90	39	11.7
Time from diagnosis to transplant in months		
<6 months	282	84.9
6–12 months	40	12.1
>12 months	10	3.0
Conditioning intensity		
BuCy/TBICy	332	100.0
Donor type		
Sibling donors	125	37.7
Unrelated donors	73	22.0
Haplo-identical donors	132	39.7
Cord blood	2	0.6
HLA		
Matched HLA	181	54.5
Mismatched HLA	151	45.5

WBC, white blood count; ELN, European LeukemiaNet; MRD, minimal residual disease; HSCT, hematopoietic stem cell transplantation; CR1, first complete remission; CR2, second complete remission; KPS, Karnofsky Performance Scale; BuCy, busulfan/cyclophosphamide; TBICy, total body irradiation/cyclophosphamide; HLA, human leukocyte antigen.

^*^These patients did not enter the establishment of the final prognostic model.

### Gene Mutations in the 332 Acute Myeloid Leukemia Patients

Among all the patients (n = 332), oncogenic mutations were identified in 37 genes, and *FLT3-ITD* was the most frequently mutated gene (74/332, 22.3%), followed by *NRAS* (68/332, 20.5%), *CEBPA* (64/332, 19.3%), *NPM1* (53/332, 16.0%), *IDH1/2* (46/332, 13.9%), *WT-1* (45/332, 13.6%), *KIT* (44/332, 13.3%), *DNMT3A* (41/332, 12.3%), and *FLT3-TKD* (37/332, 11.1%) (**Figure 1**). In total, 299 of 332 patients (90.1%) had at least one oncogenic point mutation; the median number of mutations per patient was 3 (range, 0–11), and the median number of mutated genes was 2 (range, 0–7). Furthermore, cytogenetic studies identified abnormalities in 179 patients (53.9%). Combining gene mutations and cytogenetic changes, 117 patients (35.5%) were in the favorable group, 106 patients (31.7%) were in the intermediate group, and 109 patients (32.8%) were in the adverse group according to the ELN-2017 risk classification.

### Candidate Variable Selection for Cumulative Incidence of Relapse in the RSF Model

The clinical variables and 37 gene mutations were all used to train the RSF models. After selection of the MD and VIMP (**Supplementary Figures S2, S3**), four gene mutations (*TP53*, *FLT3-ITD*, *PHF6*, and *NOTCH1*) and three other variables (cytogenetic abnormality, disease status, and pre-MRD) were chosen as candidates for predicting disease relapse after transplantation. More details of variable selection in the RSF method are described in the **Supplementary Data**.

### Univariate Analysis of Candidate Predictors for Cumulative Incidence of Relapse in the Training Cohort

A total of 12 patients who had missing pre-MRD data were excluded from the last model. Therefore, 320 of the 332 patients treated with the MA regimen were included in the last predicted model (**Supplementary Figure S1**). In the univariate analysis of these 320 patients, for the mutation of *FLT3-ITD*, the cumulative incidence of recurrence in patients with a high ratio mutation (≥0.5, n = 36) was higher than those in the *FLT3-ITD* ^low ratio^ (<0.5, n = 37) or *FLT3-ITD^Neg^* groups (n = 247) (p < 0.05; **Supplementary Table S4**), whereas there was no significant difference in the CIR between patients with an *FLT3-ITD* ^low ratio^ mutation and those in the *FLT3-ITD^Neg^* group [subdistribution hazard ratio (SHR): 0.91, p = 0.797]. Moreover, in regard to the disease stage before transplantation, patients with CR1 were chosen as the reference category, and similar SHRs of 2.99 and 2.94 were presented for patients with >CR1 and NR (p = 0.001). Based on these observations, *FLT3-ITD* ^Neg^ and the *FLT3-ITD* ^low ratio^, patients who received allo-HSCT underwent >CR1 and NR were combined as a subgroup.

### Independent Predictors for Cumulative Incidence of Relapse in the Training Cohort

Based on backward elimination, *PHF6* mutations were excluded from the prediction model. The results (**Table 2**) demonstrated that disease stage pretransplant (>CR1 *vs*. CR1), pre-MRD (pos *vs*. neg), *FLT3-ITD* (high ratio *vs*. neg or low ratio), *TP53* mutation (pos *vs*. neg), and cytogenetic classification (adverse *vs*. intermediate *vs*. favorable) were independent risk factors for AML recurrence after allo-HSCT (p < 0.05). No multicollinearity or interactions were found in the prediction model (**Table 2** and **Supplementary Table S5**).

**Table 2:** Multivariable analysis of factors associated with CIR of the 320-patient primary cohort.

	Subdistribution hazard ratio (SHR)	95% Confidence Interval	p-value	VIF
Pre-MRD (Pos *vs*. Neg)	1.98	1.16~3.36	0.012	1.366
Disease status pre-HSCT (>CR1 *vs*. CR1)	1.90	1.03~3.51	0.040	1.594
*TP53* mutation (Pos *vs*. Neg)	3.47	1.68~7.15	0.001	1.260
FLT3-ITD mutation (high ratio *vs*. Neg or low ratio)	2.07	1.08~3.95	0.028	1.153
Cytogenetic abnormality				
Favorable	1.0*	1.0*		
Intermediate	3.37	1.41~8.02	0.006	3.499
Adverse	3.48	1.27~9.53	0.015	4.027

^＊^indicates reference category; VIF, variance inflation factor; CR1, first complete remission; MRD, minimal residual disease; HSCT, hematopoietic stem cell transplantation; CIR, cumulative incidence of relapse.

### Construction, Calibration, and Validation of the Nomogram

**Figure 2:**
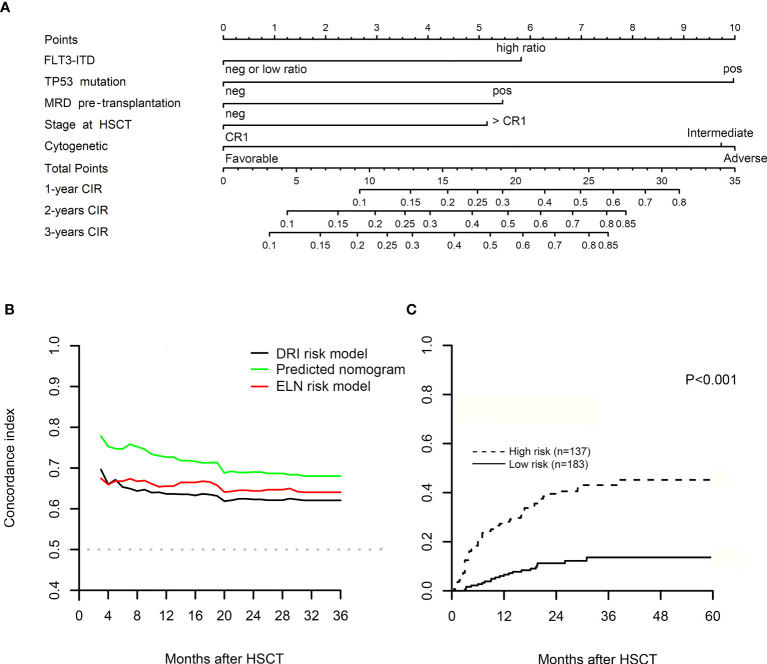
**(A)** Competing risk nomogram for predicting leukemia recurrence in acute myeloid leukemia (AML) patients with myeloablative conditioning (MAC) regimen in 1, 2, and 3 years after allogeneic hematopoietic stem cell transplantation (allo-HSCT). In the nomogram, the sum of five variable points is located on the axis of total points, and a line is drawn downward to the cumulative incidence of relapse (CIR) axes to determine the probability of 1-, 2-, and 3-year CIR. **(B)** The diagrams of the concordance index (C-index) after allo-HSCT. **(C)** The plot of CIR after risk stratification of the nomogram in the training cohort. By using the scoring system from the nomogram, the median cutoff value was adopted to divide the training cohort into two subgroups after ranking by total score (score: from 0 to 9.73 and from 9.73 to 30.58). Gray’s test was used to examine the difference in CIR between groups. MRD, minimal residual disease; ELN, European LeukemiaNet recommendations; DRI, refined Disease Risk Index.

**Figure 2A** shows the nomogram for CIR that integrated all the predictors in the last multivariate model. The plot of the time-dependent C-index is presented in **Figure 2B**, and the C-index values at 6, 12, 18, and 24 months for nomogram prediction were 0.754, 0.730, 0.715, and 0.690, respectively. Refined DRI and ELN-2017 risk recommendations were also performed to reclassify 320 patients into different risk groups, and the comparison of C-index was used to measure the discrimination performance of the predicted models. At the same time points, the C-index values were 0.653, 0.637, 0.635, and 0.623 for refined DRI risk model, and ELN-2017 risk model showed equality values of 0.668, 0.656, 0.666, and 0.644, respectively. Although the C-index of nomogram prediction presented a few higher, no statistical difference was found at the level of 5% (**Supplementary Table S6**).

**Figure 3:**
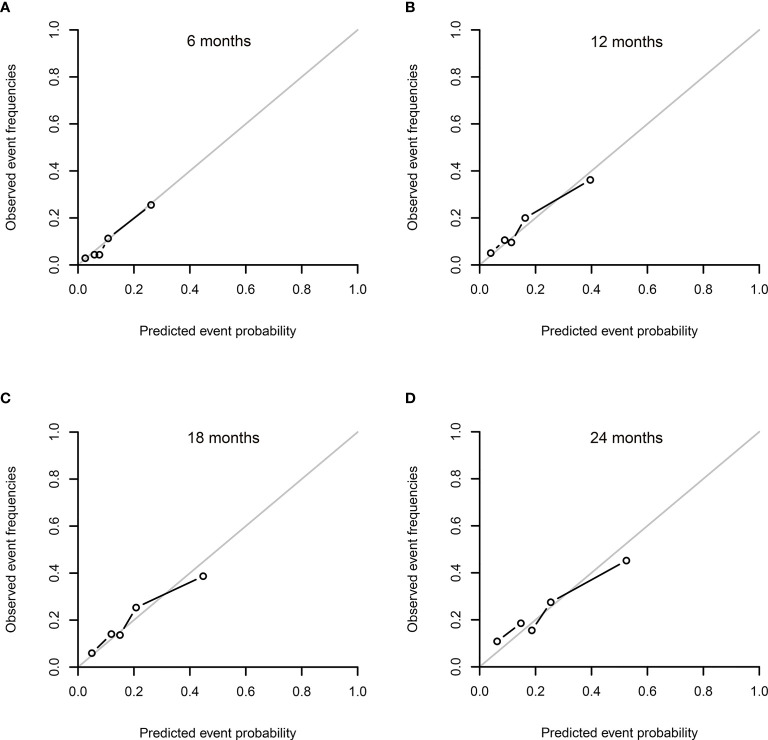
Calibration plots for the cumulative incidence of relapse (CIR) in the internal validation at 6 **(A)**, 12 **(B)**, 18 **(C)**, and 24 **(D)** months after allogeneic hematopoietic stem cell transplantation (allo-HSCT). The calibration curves were plotted for the internal validation using a bootstrapping method with 1,000 resamples. (The gray line represents perfect equality between the observed and predicted probabilities. The closer to the gray line, the more excellent agreement was revealed between the probabilities of prediction and actual observations).

The values of the ICI and E50 (**Table 3**) and calibration curves presented good agreement between the actual observation and the nomogram prediction at different time points after allo-HSCT (**Figures 3A–D**). Especially at 6 months after allo-HSCT, slight discrepancy was observed between the actual observation and the nomogram prediction, with values of 0.008 and 0.006 for the ICI and E50, respectively.

**Table 3:** ICI, E50, and E90 in the internal validation of 320-patient primary cohort using bootstrapping method.

Time after transplantation	ICI	E50	E90
6 months	0.008	0.006	0.013
12 months	0.055	0.040	0.116
18 months	0.094	0.073	0.188
24 months	0.136	0.111	0.256

ICI, integrated calibration index.

### Risk Stratification of the Nomogram in the Training Cohort

**Figure 4:**
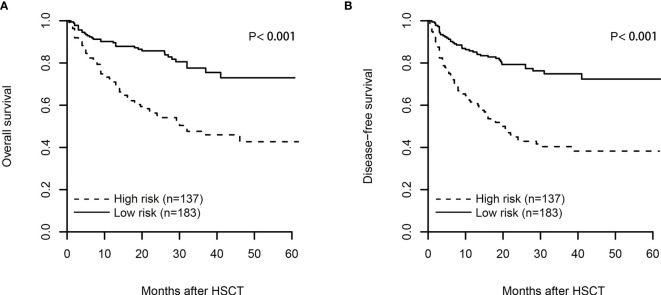
The plot of overall survival (OS, **A**) and disease-free survival (DFS, **B**) after risk stratification of the nomogram in the training cohort. By using the scoring system from the nomogram, the median cutoff value was adopted to divide the training cohort into two subgroups after ranking by total score (score: from 0 to 9.73 and from 9.73 to 30.58). The log-rank test was used to examine the difference between groups.

By using the scoring system from the nomogram, we calculated a risk score for patients in the training cohort who had no missing data on any of the five variables (n = 320), with a median score of 9.73 (range from 0 to 30.58). The curve of restricted cubic spline showed a linear relationship (**Supplementary Figure S5B**, p for nonlinearity = 0.510), and the median cutoff value was adopted to divide all patients into two subgroups after ranking by the total score (score: from 0 to 9.73 and 9.73 to 30.58). The estimated probabilities of 2-year CIR (**Figure 2C**), 2-year DFS (**Figure 4B**), and 2-year OS (**Figure 4A**) were 40.5%, 42.9%, and 55.2% in high-risk patients with scores >9.73 and 11.2%, 79.3%, and 85.8% in low-risk patients with scores ≤9.73, and the differences between these two groups were significant (p < 0.001). However, no difference was seen in the probability of 2-year NRM between high-risk and low-risk patients (16.5% *vs*. 9.5%, p = 0.256; **Supplementary Figure S5C**).

### Clinical Use

**Figure 5:**
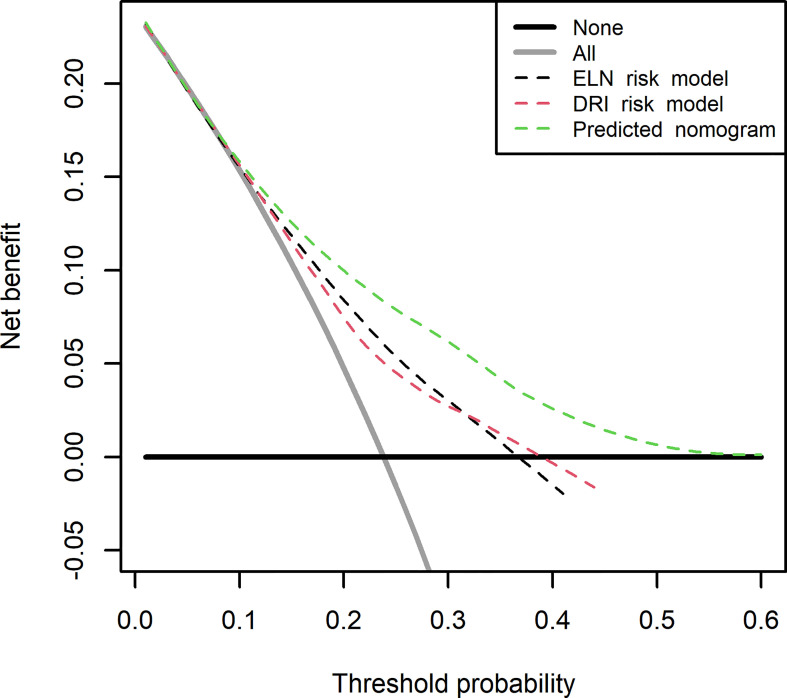
Decision curve analysis for the predicted nomogram and the model with Disease Risk Index (DRI) and European LeukemiaNet (ELN)-2017 risk recommendation. The green line represents the predicted nomogram. The red and black line represents the predicted model with DRI and ELN-2017 risk recommendation. Solid gray line indicates net benefit when all patients are considered as disease relapse after allogeneic hematopoietic stem cell transplantation (allo-HSCT). Bold black line indicates net benefit when all patients are not considered as developing disease relapse. The predicted nomogram showed the highest net benefit when threshold is higher than 6%.

The DCA for the predicted nomogram and two other risk models (refined DRI and ELN-2017) is presented at time of 24 months after allo-HSCT in **Figure 5**. On the basis of three predicted models, net benefits showed overlapping curves at a threshold lower than 6%. Range from a threshold of 6%–60%, several improvements were presented for nomogram prediction than the two other risk models. If the threshold probability of a doctor or patient is higher than 6%, using the nomogram to predict AML relapse after HSCT could add more benefits than either the scheme of treat-none or treat-all-patients.

## Discussion

It has been well recognized in population studies that the survival of AML patients with allo-HSCT can vary. Pivotal historical studies have shown that remission status at the time of transplant, cytogenetics, age, HLA matching, performance status, and comorbidity indices have prognostic utility (18, 19, 22, 23). Armand et al. developed a DRI to predict the outcome for patients undergoing allogeneic stem cell transplantation and validated the index in a large cohort of the CIBMTR database. However, it should be noted that only cytogenetics was involved in the DRI for patients undergoing allogeneic stem cell transplantation.

Genetic abnormalities are powerful prognostic factors, and somatic mutations have been linked to survival outcomes after allo-HSCT, such as mutations in the *NPM1* and *FLT3* genes (24, 25). The Acute Leukemia Working Party of the European Society for Blood and Marrow Transplantation (EBMT) reported a retrospective registry analysis of 702 adults with CN-AML undergoing HSCT in CR1. These researchers found that *FLT3-ITD* was the decisive molecular marker for outcome after HSCT for CN-AML in CR1 (15). MRD monitoring for *NPM1* mutations has proven to be highly predictive for relapse in AML patients treated with or without allo-HSCT. However, it is not clear to date from clinical data whether AML patient subgroups characterized by different combinations of molecular markers, such as *IDH1/2*, *TET2*, *DNMT3A*, and *ASXL1*, have different outcomes after allo-HSCT, since the number of transplanted patients in reported studies are relatively small. Moreover, it is important to generate a simple prediction model including the genetic abnormalities and disease status for AML patients with allo-HSCT.

In this study, we used targeted NGS test to identify gene mutations and gene rearrangements in 332 AML patients who underwent the allo-HSCT. In a multivariable analysis adjusted by other confounding factors, mutations in *TP53* and *FLT3-ITD* ^high ratio^ were strongly associated with an increased probability of disease relapse. Combined with the disease status, pre-MRD, and cytogenetic abnormality, we constructed a nomogram to predict the CIR after allo-HSCT, and the time-dependent C-index at 6, 12, 18, and 24 months for CIR prediction was 0.754, 0.730, 0.715, and 0.690, respectively. However, based on these five predictors and the threshold score, except *TP53* mutation or adverse cytogenetic abnormality whose single score was higher than 9.73, it was difficult to stratify the patients with lower than two other risk factors into different risks, which may limit the usability of this nomogram. And the heterogeneity of this group of patients may benefit from other predictable markers, such as other gene mutations. Future studies on large samples may increase weights of other gene mutations, especially for significant mutations but with low frequency, and combination with these new gene mutations may improve the discrimination and applicability of the predicted model for AML relapse after transplantation. Moreover, independent external validation data will still be required to validate the nomogram models in the future, making the models more reliable.

Using NGS for MRD detection is appealing because its flexibility allows the use of almost every mutated gene as an MRD marker. Multiple studies have demonstrated a strong correlation between NGS-based MRD status and the subsequent risk of relapse in AML patients, especially for a persisting positive MRD status at various posttreatment time points (26–28). In this study, NGS-MRD was not included in the nomogram for predicting CIR, and we are now carrying out a clinical research investigating the predictive value of NGS-MRD for AML patients with allo-HSCT. The flow-MRD approach combined with the NGS-MRD approach may help to refine transplant and posttransplant management in AML patients.

To the best of our knowledge, our study is the first to construct a nomogram of AML relapse after allo-HSCT in patients receiving an MA regimen. Nevertheless, our study has several limitations. First, this retrospective analysis was based on data from a single institution, and the total number of cases was not large enough. This finding may be due to the lower morbidity of AML, especially for patients who both received allo-HSCT and were tested by targeted exome sequencing and RNA sequencing at diagnosis. The economic cost of NGS should be considered. Meanwhile, a prospective study and the results from other centers are necessary to confirm the reliability of this recurrent nomogram. Second, the nomogram achieved a favorable predictive accuracy, especially in the early term after allo-HSCT (18 months). However, as time passed after allo-HSCT, a stepwise depletion of C-index values was seen in our cohort, and discrimination from the C-index at different times suggested that this model is less concordant at later time points than in earlier time points after transplantation. Third, one important limitation of a nomogram is its inability to incorporate confidence intervals for the individual variables into a final summed score. In our results, *TP53* mutation was awarded the maximal 10 points from the nomogram, corresponding to its high SHR for relapse, but the confidence interval was quite large due to its low number. These factors may misestimate and limit the interpretability of the true individual-level risk associated with *TP53* mutations. An increase in the sample size may narrow the confidence intervals and obtain more precise estimates in future studies. Meanwhile, we did not perform external validation due to limited patient numbers, and further study may also improve this limitation. Finally, because this model was based on both clinical and laboratory data, precautions should be taken for the definition and results of pre-MRD and NGS at diagnosis when interpreting this recurrent nomogram. In the future, better markers, such as quantification of somatic mutation, might further improve the prediction model.

The most common argument of predicted nomogram is clinical use; interpretation should be made whether clinical intervention is necessary. DCA offers a straightforward insight into clinical outcomes. To evaluate the clinical usefulness, net benefit from our predicted nomogram was verified to improve consequences. The DCA indicated that if the threshold probability of a doctor or patient is higher than 6%, using the nomogram in our study to predict AML relapse after allo-HSCT could add more benefits than either the scheme of treat-none or treat-all-patients.

In conclusion, with NGS, we constructed a predictive nomogram including disease status pretransplant, pre-MRD, cytogenetic risk classification, and *TP53* and *FLT3-ITD* ^high ratio^ mutations for CIR in AML patients after allo-HSCT. Our findings strongly suggest that molecular aberrations should be considered to optimize the current prediction models for the recurrence of AML after allo-HSCT.

## Data Availability Statement

The data presented in the study are deposited in the GSA-Human repository, accession number is: HRA001325. Please access it from the following link: https://bigd.big.ac.cn/gsa-human/browse/HRA001325.

## Ethics Statement

The studies involving human participants were reviewed and approved by the ethics committee of the First Affiliated Hospital of Soochow University. The patients/participants provided their written informed consent to participate in this study.

## Author Contributions

TZ, XB, SC, and YX conceived the study. TZ and XB collected the clinical samples. YX, TZ, and XB analyzed the data. HQ, XT, YH, CF, and AS provided guidance on experimental design, data analysis, and presentation of results. TZ and XB wrote the paper. CR, SC, DW, and YX revised the paper. All authors contributed to the article and approved the submitted version.

## Funding

This work has been supported by grants from the National Natural Science Foundation of China (81730003, 81870120, 81900130), the Natural Science Foundation of Jiangsu Province (BK20171205), the Social Development Project of Jiangsu Province (BE2019655), the Priority Academic Programme Development of Jiangsu Higher Education Institutions (PAPD), and the National Key Research and Development Programme (2016YFC0902800, 2017YFA0104500, 2019YFC0840604).

## Conflict of Interest

The authors declare that the research was conducted in the absence of any commercial or financial relationships that could be construed as a potential conflict of interest.

## Publisher’s Note

All claims expressed in this article are solely those of the authors and do not necessarily represent those of their affiliated organizations, or those of the publisher, the editors and the reviewers. Any product that may be evaluated in this article, or claim that may be made by its manufacturer, is not guaranteed or endorsed by the publisher.
