# Supplementary material for: Development of a Nomogram for Predicting the Cumulative Incidence of Disease Recurrence of AML After Allo-HSCT
Source: Front Oncol. 2021 Sep 27;11:732088. doi: 10.3389/fonc.2021.732088 (PMC8503644; doi:10.3389/fonc.2021.732088)
Supplement: Supplementary file 1 [file DataSheet_1.doc]

**Supplementary methods**

**Random Survival Forest (RSF)**

By means of binary decision trees, a Random Survival Forest (RSF) was constructed to screen the most important variables for survival analysis with competing risks data [1]. Decision trees of RSF were grown from bootstrap samples of two thirds of the original data, with modified Gray's log-rank splitting rule [2]. The remaining data was excluded in each bootstrap sample and called out-of-bag (OOB) data. According to the recommendation described by Ishwaran et al. [1], the main parameters were performed as follows: *ntree*=1000; *mtry*=12 (number of predictors/4); and *nodesize*=6. All function were performed by the R-package randomForestSRC. variable selection used for RSF contained minimal depth (MD) and variable importance (VIMP).

**MD**

After the RSF construction, MD was used to assess how informative variables for disease relapse, which was described in detail by Ishwaran et al. [3]. Within each tree of the RSF, the distance was determined from the closest node to the root node when the respective variable splits first. Based on the relative distance to the root node of forest, the depth (node levels) were recorded. Over all trees within the RSF, MD was ranking to measure the importance of variables by averaging the depth of the variable first split. The lower the values of MD, the more predictability a variable possessed. Using the mean value of the MD distribution [3], values of MD smaller than this threshold were classified as important variables and chosen for further analysis. From the results of **Figure S2,** eleven variables had lower MD than the threshold (9.68), including 4 gene mutations (*TP53, FLT3-ITD, PHF6* and *NOTCH1*) and 7 other factors (cytogenetic abnormality, age, disease status and pre-MRD, Donor-type, blood-type and gender-type matching).

**VIMP**

According to the impact on predictive ability of the RSF, VIMP was another approach to select the importance of variables. No predictive accuracy is improved when VIMP close to zero, and including a negative VIMP indicate the reduction of predictive accuracy. A large VIMP value indicates an improvement of the predictive accuracy in the RSF model. After selection of the VIMP (**Figure S3**), donor-type, age, matching of blood-type and gender-type were ignored with lower VIMP values. Finally, 4 gene mutations (*TP53, FLT3-ITD, PHF6* and *NOTCH1*) and 3 other variables (cytogenetic abnormality, disease status and pre-MRD) were included as candidates for predicting disease relapse after allogeneic haematopoietic stem cell transplantation (allo-HSCT).

**Univariate analysis of candidate variables for predicting disease relapse**

Univariate analysis (**Table S4**) was performed in candidates for predicting leukemia recurrence, containing 4 gene mutations (*TP53*, *FLT3-ITD*, *PHF6* and *NOTCH1*) and 3 other variables (cytogenetic abnormality, disease status and pre-MRD). Moreover, mutation of *NPM1* was also tested in univariate analysis, with a VIMP value of 0.005 and a MD value of 10.44, but without any statistic difference. Considering that *KIT* mutation has a great influence on AML with core-binding factor (*CBF*) transcript, we reclassified the cytogenetic abnormality into four groups. There was no significant difference in AML recurrence between *CBF*-AML with and without *KIT* mutation (**Table S4,** *P* > 0.05). Moreover, in the four classifications, abnormally wide confidence intervals were presented both in groups with Intermediate and adverse cytogenetic abnormality. To get higher statistical power, *CBF*-AML with and without *KIT* mutation were combined as a subgroup.

**Variable Interactions**

In the last multivariable prediction model for leukemia recurrence after transplantation (**Table 2**), pairwise interactions among interest variables were detected and screened by MD method first [3, 4]. **Figure S4** showed the interaction plot for five interest variables. Variables with higher values of MD indicated lower interaction effects with target variable marked in red. Scanning across the plots of each target variables, the covariate with the lowest MD value may be the most possibly accompanied with interaction effect, as it typically split close to the root node. The interaction and subgroup analyses were also tested and confirmed between *TP53, FLT3-ITD,* pre*-*MRD, diseases status pre-HSCT and cytogenetic abnormality (**Table S5**), but no statistic difference were found (*P* > 0.05).

**Supplementary Tables**

| Patients after excluded unknown pre-MRD (n=320) | | No. patients | Definition of pre-MRD |
| --- | --- | --- | --- |
| Fusion Gene Transcripts for MRD Assessment (n=98) | recurrent fusion genes(-) | 58 | pre-MRDneg |
|  | recurrent fusion genes(+) and MFC-MRD(-) | 10 | pre-MRDpos |
|  | recurrent fusion genes(+) and MFC-MRD(+) | 30 | pre-MRDpos |
| MFC-MRD and/or WT1 expression for MRD Assessment (n=222) | MFC-MRD(+) | 43 | pre-MRDpos |
|  | MFC-MRD(-) and WT1 expression (+) | 15 | pre-MRDpos |
|  | MFC-MRD(-) and WT1 expression (-) | 112 | pre-MRDneg |
|  | MFC-MRD(-) and WT1 not performed | 52 | pre-MRDneg |

**Table S1. The pre-MRD monitoring and definition.**

**Table S2. The list of 382 known or presumed mutant genes in targeted sequencing.**

| *ABCB1* | *CEBPA* | *GRIN2A* | *MYCN* | *SETD2* | *ABCC2* |
| --- | --- | --- | --- | --- | --- |
| *ABL1* | *CEP57* | *GSTM1* | *MYD88* | *SF3B1* | *ADH1B* |
| *ABL2* | *CHEK1* | *GSTT1* | *NBN* | *SGK1* | *ALDH2* |
| *ACTB* | *CHEK2* | *HBA1* | *NCSTN* | *SH2D1A* | *ARID5B* |
| *AIP* | *CIITA* | *HBA2* | *NF1* | *SMAD2* | *BCR* |
| *AKT1* | *CKS1B* | *HBB* | *NF2* | *SMAD4* | *BIM* |
|  |  |  |  |  | *(BCL2L11)* |
| *AKT2* | *CREBBP* | *HDAC1* | *NFKBIA* | *SMARCA4* | *CDK10* |
| *AKT3* | *CRLF2* | *HDAC2* | *NKX2-1* | *SMARCB1* | *CYP19A1* |
| *ALK* | *CSF1R* | *HDAC4* | *NOTCH1* | *SMC1A* | *CYP2A6* |
| *AP3B1* | *CSF3R* | *HDAC7* | *NOTCH2* | *SMC3* | *CYP2B6*6* |
| *APC* | *CTCF* | *HGF* | *NPM1* | *SMO* | *CYP2C19*2* |
| *AR* | *CTLA4* | *HNF1A* | *NRAS* | *SOX2* | *CYP2C9*3* |
| *ARHGAP26* | *CTNNB1* | *HRAS* | *NSD1* | *SRC* | *CYP2D6*3* |
| *ARID1A* | *CUX1* | *ID3* | *NT5C2* | *SRSF2* | *CYP2D6*4* |
| *ARID2* | *CXCR4* | *IDH1* | *NTRK3* | *STAG2* | *CYP2D6*5* |
| *ASXL1* | *CYLD* | *IDH2* | *NUP98* | *STAT3* | *CYP2D6*6* |
| *ATM* | *CYP2D6* | *IGF1R* | *P2RY8* | *STAT5A* | *CYP3A4*4* |
| *ATR* | *DAXX* | *IKBKE* | *PAG1* | *STAT5B* | *CYP3A5*3* |
| *ATRX* | *DDR2* | *IKZF1* | *PAK3* | *STAT6* | *DHFR* |
| *AURKA* | *DICER1* | *IKZF2* | *PALB2* | *STIL* | *ENOSF1* |
| *AURKB* | *DNM2* | *IKZF3* | *PAX5* | *STK11* | *ERCC1* |
| *AXIN1* | *DNMT3A* | *IL7R* | *PBRM1* | *STMN1* | *ETV1* |
| *AXL* | *DOT1L* | *INPP4B* | *PC* | *STX11* | *ETV4* |
| *B2M* | *DPYD* | *INPP5D* | *PDCD1* | *STXBP2* | *ETV5* |
| *BAP1* | *DUSP2* | *IRF1* | *PDCD1LG2* | *SUFU* | *EWSR1* |
| *BARD1* | *EBF1* | *IRF8* | *PDGFRA* | *SUZ12* | *FCGR2B* |
| *BCL10* | *ECT2L* | *JAK1* | *PDGFRB* | *TBL1XR1* | *GNA11* |
| *BCL11B* | *EED* | *JAK2* | *PDK1* | *TCF3* | *GNAQ* |
| *BCL2* | *EGFR* | *JAK3* | *PHF6* | *TCL1A* | *GNAS* |
| *BCL2L1* | *EGR1* | *JARID2* | *PHOX2B* | *TEK* | *GSTP1* |
| *BCL2L2* | *EP300* | *JUN* | *PIK3CA* | *TET2* | *HNF1B* |
| *BCL6* | *EPCAM* | *KDM2B* | *PIK3CD* | *TGFBR2* | *MECOM* |
| *BCL7A* | *EPHA3* | *KDM5A* | *PIK3R1* | *TLE1* | *MLL* |
| *BCOR* | *ERBB2* | *KDR* | *PIK3R2* | *TLE4* | *MTHFR* |
| *BCORL1* | *ERBB3* | *KIT* | *PIM1* | *TNFAIP3* | *MYCL1* |
| *BIRC3* | *ERBB4* | *KMT2A* | *PMS1* | *TNFRSF11A* | *NQO1* |
| *BLM* | *ERCC2* | *KMT2B* | *PMS2* | *TNFRSF14* | *NRG1* |
| *BMPR1A* | *ERCC3* | *KMT2C* | *POT1* | *TNFRSF17* | *NTRK1* |
| *BRAF* | *ERCC4* | *KMT2D* | *PPP2R1A* | *TOP1* |  |
| *BRCA1* | *ERCC5* | *KRAS* | *PRDM1* | *TOP2A* |  |
| *BRCA2* | *ERG* | *LEF1* | *PRF1* | *TP53* |  |
| *BRD4* | *ETS1* | *LMO1* | *PRKAR1A* | *TP63* |  |
| *BRIP1* | *ETV6* | *LYN* | *PTCH1* | *TPMT* |  |
| *BTG2* | *EZH2* | *LYST* | *PTEN* | *TRAF2* |  |
| *BTK* | *FANCA* | *MAF* | *PTPN11* | *TRAF3* |  |
| *BTLA* | *FANCC* | *MAFB* | *PTPN2* | *TRAF5* |  |
| *BUB1B* | *FANCD2* | *MALT1* | *PTPN6* | *TSC1* |  |
| *CALR* | *FANCE* | *MAP2K1* | *PTPRO* | *TSC2* |  |
| *CBL* | *FANCF* | *MAP2K2* | *RAD21* | *TSHR* |  |
| *CCND1* | *FANCG* | *MAP2K4* | *RAD50* | *TTF1* |  |
| *CCND2* | *FANCL* | *MAP3K1* | *RAD51* | *TUBB3* |  |
| *CCND3* | *FAT1* | *MCL1* | *RAF1* | *U2AF1* |  |
| *CCNE1* | *FBXO11* | *MDM2* | *RARA* | *UGT1A1* |  |
| *CCT6B* | *FGFR1* | *MDM4* | *RASGEF1A* | *UNC13D* |  |
| *CD22* | *FGFR2* | *MED12* | *RB1* | *VEGFA* |  |
| *CD274* | *FGFR3* | *MEF2B* | *RECQL4* | *VHL* |  |
| *CD58* | *FGFR4* | *MEN1* | *RELN* | *WHSC1* |  |
| *CD70* | *FH* | *MET* | *RET* | *WT1* |  |
| *CDA* | *FIP1L1* | *MGMT* | *RICTOR* | *XIAP* |  |
| *CDC73* | *FLCN* | *MITF* | *ROS1* | *XPC* |  |
| *CDH1* | *FLT1* | *MLH1* | *RPTOR* | *XPO1* |  |
| *CDK12* | *FLT3* | *MPL* | *RRM1* | *YAP1* |  |
| *CDK4* | *FLT4* | *MRE11A* | *RUNX1* | *ZAP70* |  |
| *CDK6* | *FOXO1* | *MSH2* | *RUNX1T1* | *ZRSR2* |  |
| *CDK8* | *FOXO3* | *MSH3* | *SBDS* |  |  |
| *CDKN1B* | *GADD45B* | *MSH6* | *SDHB* |  |  |
| *CDKN1C* | *GATA1* | *MTOR* | *SDHC* |  |  |
| *CDKN2A* | *GATA2* | *MUTYH* | *SDHD* |  |  |
| *CDKN2B* | *GATA3* | *MYC* | *SERP2* |  |  |
| *CDKN2C* | *GNA13* | *BAGE2* | *SETBP1* |  |  |

**Table S3. Point Assignment and Predicted Score**

| Variable | Score | Total score | Estimated 2-Year CIR |
| --- | --- | --- | --- |
| *FLT3-ITD* mutation |  | 27.54 | 0.85 |
| Neg or low ratio | 0 | 26.23 | 0.80 |
| High ratio | 5.82 | 23.90 | 0.70 |
| Cytogenetic abnormality |  | 21.71 | 0.60 |
| Favorable | 0 | 19.47 | 0.50 |
| Intermediate | 9.73 | 17.03 | 0.40 |
| Adverse | 10 | 14.15 | 0.30 |
| *TP53* mutation |  | 12.42 | 0.25 |
| Neg | 0 | 10.39 | 0.20 |
| Pos | 9.97 | 7.84 | 0.15 |
| Pre-MRD |  | 4.37 | 0.10 |
| Neg | 0 |  |  |
| Pos | 5.46 |  |  |
| Disease status pre-HSCT |  |  |  |
| CR1 | 0 |  |  |
| >CR1 | 5.15 |  |  |

**Table S4. Univariate analysis of candidate variables for disease relapse of the 320 primary cohort.**

|  | **Subdistribution hazard ratio (SHR)** | **95 % Confidence Interval (95% CI)** | **P value** |
| --- | --- | --- | --- |
| Cytogenetic abnormality |  |  |  |
| Favorable (n=76) | 1.0^＊^ | 1.0^＊^ |  |
| Intermediate (n=189) | 3.63 | 1.55~8.50 | 0.003 |
| Adverse (n=55) | 5.17 | 2.06~12.94 | 0.001 |
| Cytogenetic abnormality |  |  |  |
| Favorable with *KIT* ^neg^ (n=42) | 1.0^＊^ | 1.0^＊^ |  |
| Favorable with *KIT* ^Pos^ (n=34) | 6.96 | 0.80~60.8 | 0.079 |
| Intermediate (n=189) | 12.47 | 1.68~92.7 | 0.014 |
| Adverse (n=55) | 17.84 | 2.33~136.6 | 0.006 |
| Pre-MRD (Pos vs. Neg) | 2.36 | 1.50~3.71 | <0.001 |
| Disease status pre-HSCT |  |  |  |
| CR1 (n=264) | 1.0^＊^ | 1.0^＊^ |  |
| >CR1 (n=20) | 2.99 | 1.58~5.66 | 0.001 |
| NR (n=36) | 2.94 | 1.56~5.55 | 0.001 |
| *TP53* mutation (Pos vs. Neg) | 4.51 | 2.25~9.05 | <0.001 |
| *NOTCH1* mutation (Pos vs. Neg) | 3.57 | 1.06~12.06 | 0.041 |
| *PHF6* mutation (Pos vs. Neg) | 3.87 | 1.65~9.06 | 0.002 |
| *FLT3-ITD* mutation |  |  |  |
| Negative (n=247) | 1.0^＊^ | 1.0^＊^ |  |
| Low ratio (n=37) | 0.91 | 0.42~1.94 | 0.797 |
| High ratio (n=36) | 2.94 | 1.64~5.26 | <0.001 |
| *NPM1* mutation (Pos vs. Neg) | 1.42 | 0.81~2.48 | 0.222 |

^＊^ indicate Reference category

|  |
| --- |

**Table S5. Multivariable and stratified analyses of interest variables for disease relapse of the 320 primary cohort.**

|  | **N(%)** | **N(%)** | **95%CI** | **P value** | **P for Interaction** |
| --- | --- | --- | --- | --- | --- |
|  | *TP53* mutation | |  |  |  |
|  | Negative | positive |  |  |  |
| Cytogenetic abnormality |  |  |  |  | 0.700 |
| Favorable | 76 (100.0%) | 0 (0.0%) | NE | NE |  |
| Intermediate | 185 (98.0%) | 4 (2.0%) | 4.45 (1.44~13.75) | 0.010 |  |
| Adverse | 50 (90.9%) | 5 (9.1%) | 4.25 (1.49~12.16) | 0.007 |  |
|  | *FLT3-ITD* mutation | |  |  |  |
|  | Negative | Positive |  |  |  |
| Cytogenetic abnormality |  |  |  |  | 0.103 |
| Favorable | 75 (98.7%) | 1 (1.3%) | NE | NE |  |
| Intermediate | 161 (85.2%) | 28 (14.8%) | 2.85 (1.36~5.97) | 0.006 |  |
| Adverse | 48 (87.3%) | 7 (12.7%) | 0.99 (0.18~5.57) | 0.993 |  |
|  | Pre-MRD | |  |  |  |
|  | Negative | Positive |  |  |  |
| Cytogenetic abnormality |  |  |  |  | 0.730 |
| Favorable | 48 (63.2%) | 28 (36.8%) | 4.11 (0.79~21.50) | 0.094 |  |
| Intermediate | 143 (75.7%) | 46 (24.3%) | 1.43 (0.67~3.05) | 0.350 |  |
| Adverse | 31 (56.4%) | 24 (43.6%) | 3.33 (1.03~10.80) | 0.045 |  |
|  | Status pre-HSCT | |  |  |  |
|  | CR1 | >CR1 |  |  |  |
| Cytogenetic abnormality |  |  |  |  | 0.055 |
| Favorable | 67 (88.2%) | 9 (11.8%) | 1.74 (0.31~9.75) | 0.531 |  |
| Intermediate | 158 (83.6%) | 31 (16.4%) | 3.16 (1.55~6.45) | 0.002 |  |
| Adverse | 39 (70.9%) | 16 (29.1%) | 0.73 (0.21~2.55) | 0.623 |  |

NE indicate no enough events, For *TP53* mutation and cytogenetic abnormality, the model was adjusted by *FLT3-ITD* mutation, pre-MRD and disease status pre-HSCT; for *FLT3-ITD* mutation and cytogenetic abnormality, the model was adjusted by *TP53* mutation, pre-MRD and disease status pre-HSCT; for MRD and cytogenetic abnormality, the model was adjusted by disease status pre-HSCT, mutation of *TP53* and *FLT3-ITD*; for disease status pre-HSCT and cytogenetic abnormality, the model was adjusted by pre-MRD, mutation of *TP53* and *FLT3-ITD*.

**Table S6. Comparison of C-index in pairing time after HSCT between mutation model and risk of ELN, DRI model.**

|  | **P value** | | | |
| --- | --- | --- | --- | --- |
|  | Mutation model  (6 months) | Mutation model  (12 months) | Mutation model  (18 months) | Mutation model  (24 months) |
| ELN risk model (6 months) | 0.637 | - | - | - |
| ELN risk model (12 months) | - | 0.595 | - | - |
| ELN risk model (18 months) | - | - | 0.926 | - |
| ELN risk model (24 months) | - | - | - | 0.517 |
| DRI risk model (6 months) | 0.675 | - | - | - |
| DRI risk model (12 months) | - | 0.266 | - | - |
| DRI risk model (18 months) | - | - | 0.116 |  |
| DRI risk model (24 months) | - | - | - | 0.165 |

ELN indicate European Leukemia Net recommendations, DRI indicate refined disease risk index.

|  |
| --- |

**Supplementary Figures**


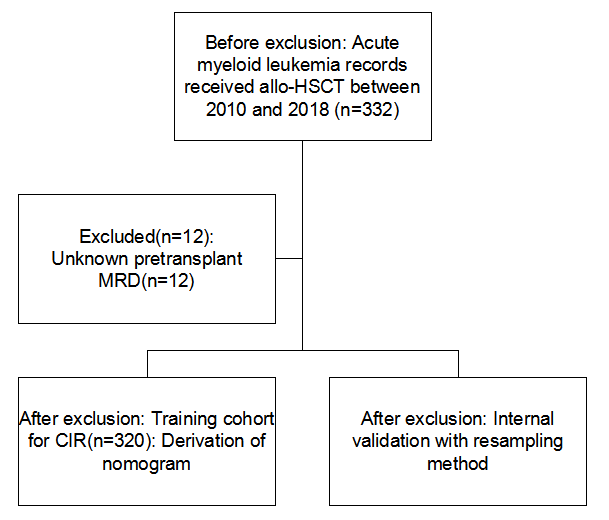


**Figure S1. The diagrams of patients selection to develop competing risk nomogram for pre-dicting leukemia recurrence after allotransplantation.**


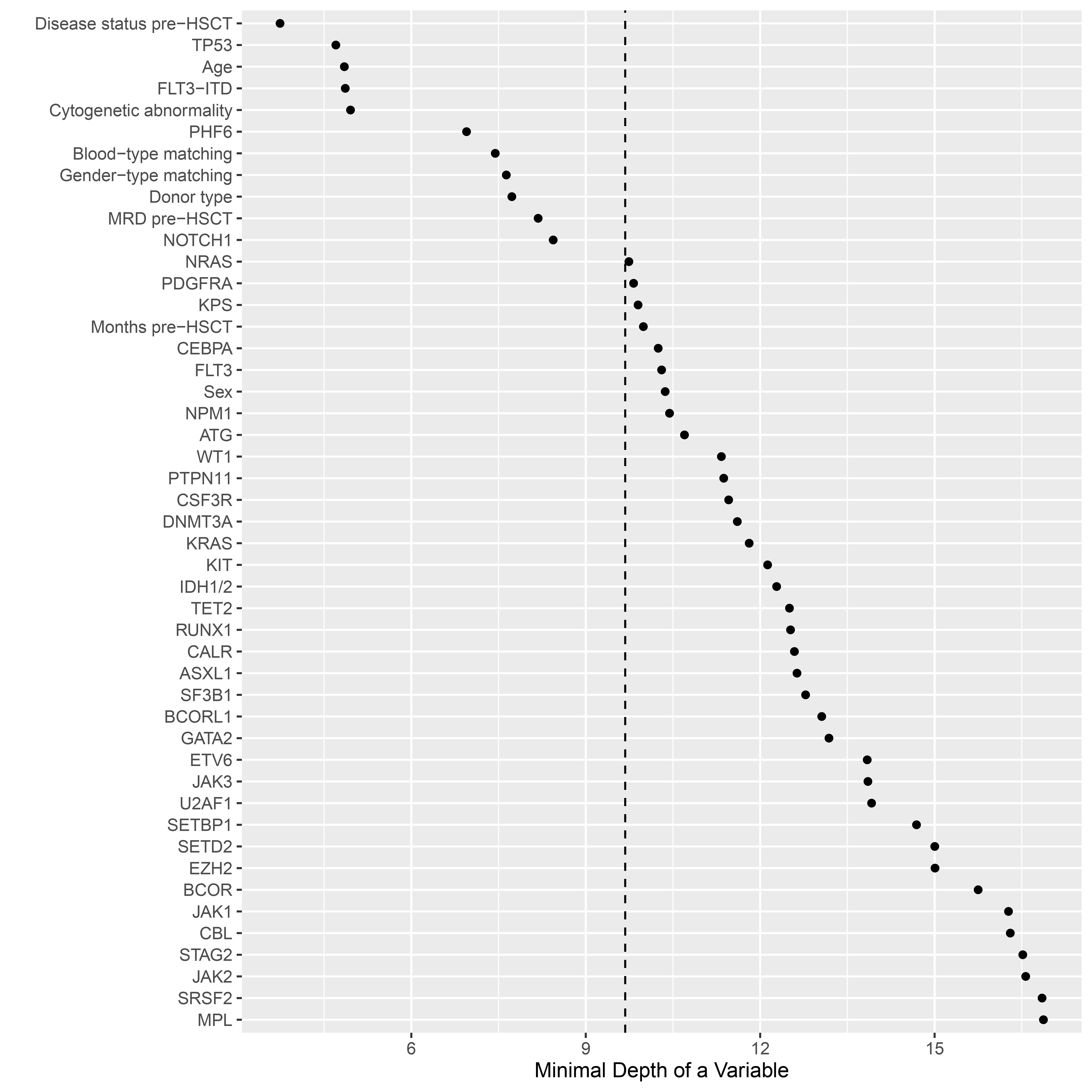
**Figure S2: Minimal Depth (MD) selection of variables in Random survival forest (RSF) model for disease relapse after transplantation. Low MD indicates important variables. The dashed line is the threshold of maximum value for variable selection.**


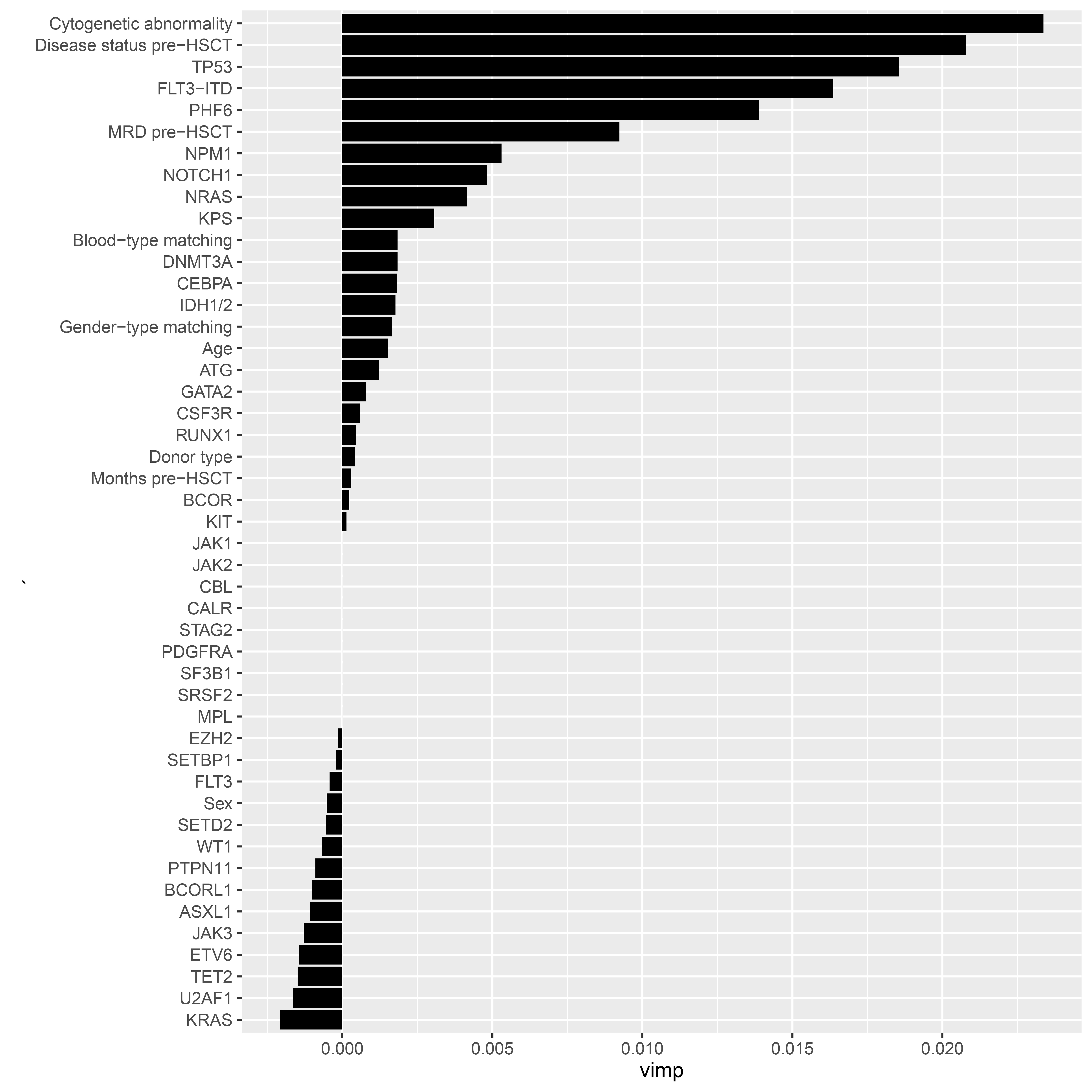
**Figure S3: Variable Importance (VIMP) of Random survival forest (RSF). Bars with values higher than zero indicate positive VIMP, and those lower than zero indicates negative VIMP. Importance is relative to positive length of bars**.


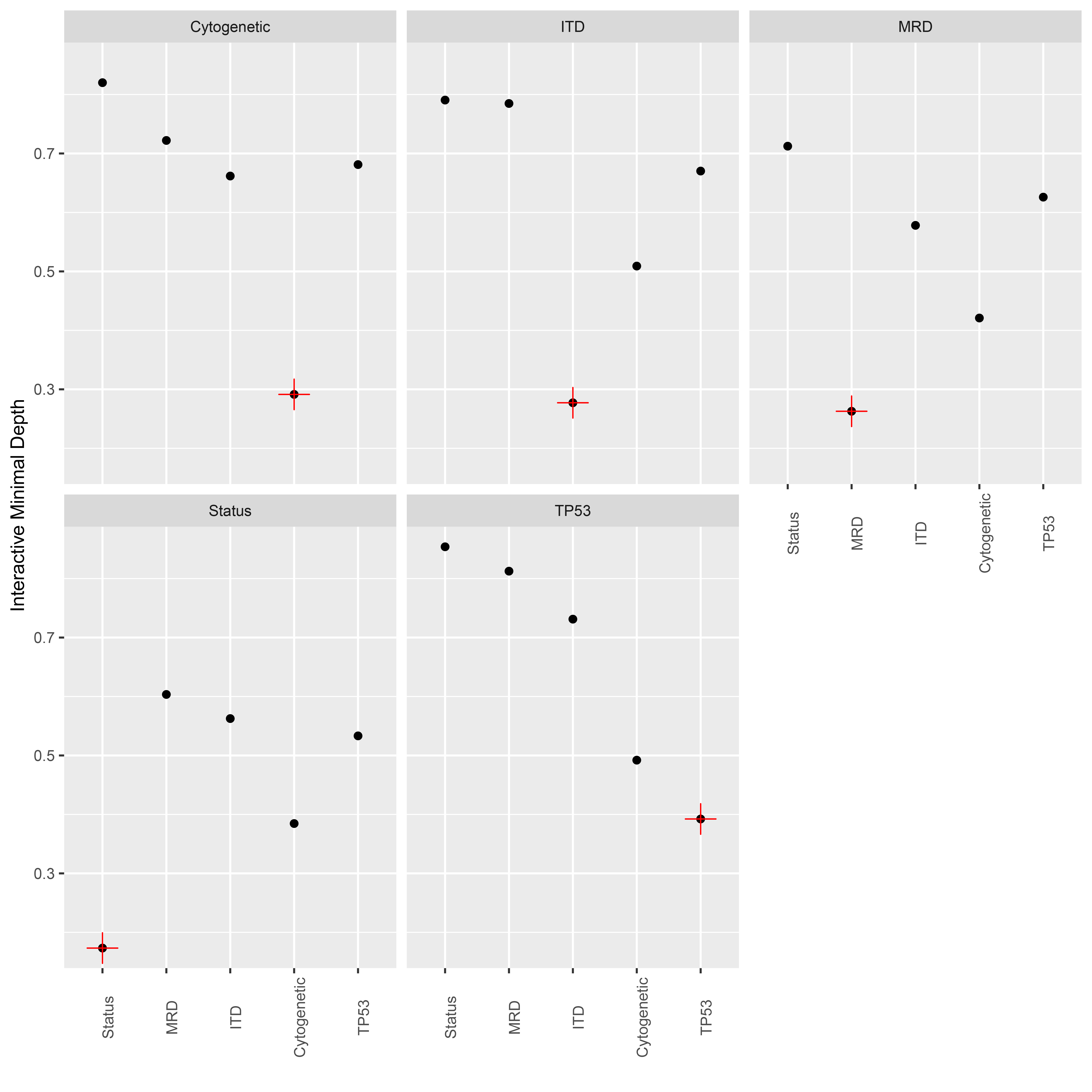


**Figure S4: Interaction plot of Minimal depth (MD) for five interest variables in the last multivariable model. Higher values of MD indicate lower interactivity with target variable marked in red. Status indicated the stage or status before allo-HSCT.**


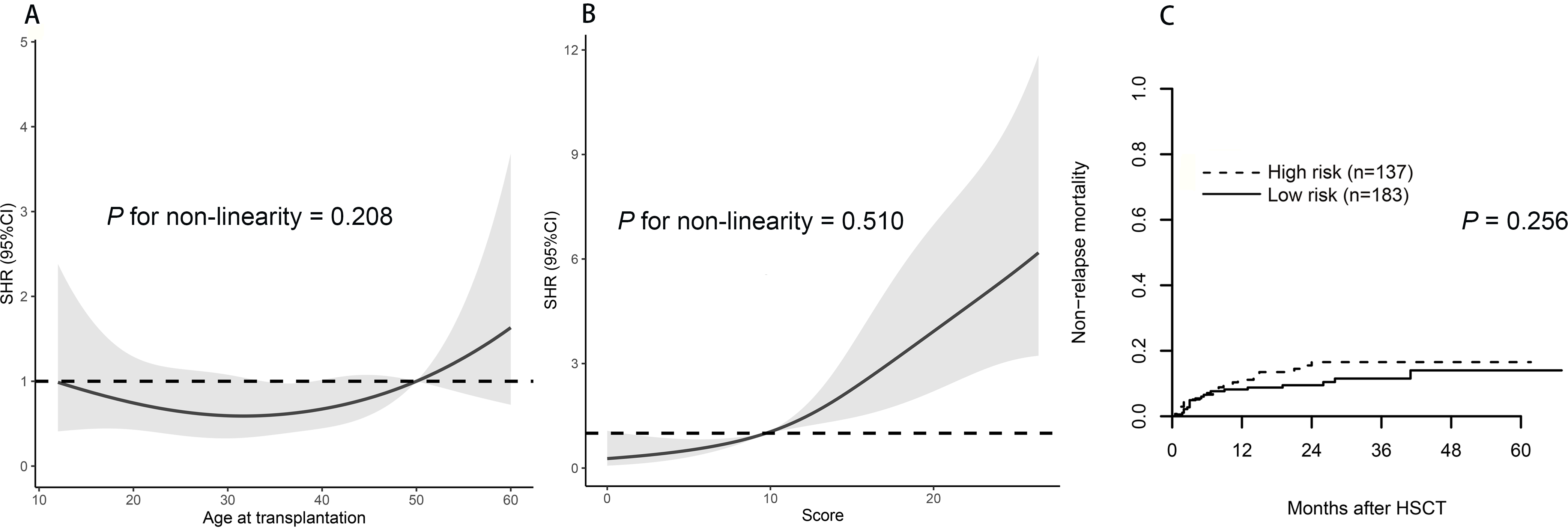


**Figure S5. (A) The linear relations between CIR and age. The restricted cubic spline was used to flexibly fit model and visualize the relation of age with the CIR in patients after allo-HSCT. (B) By using the scoring system from the nomogram, risk scores had a median score of 9.73 (range from 0 to 30.58). The curve of restricted cubic spline showed a linear relationship between score and CIR. (C) The plot of NRM after risk stratification of the nomogram in the training cohort. The median cutoff value was adopted to divide the training cohort into two subgroups after ranking by total score (score: from 0 to 9.73, and 9.73 to 30.58). The Gray’s test was used to examine the difference between groups.**

**Non-relapse mortality indicate NRM.**

**References:**

1. H. Ishwaran, T.A. Gerds, U.B. Kogalur, R.D. Moore, S.J. Gange, B.M. Lau, Random survival forests for competing risks, Biostatistics 15 (4) (2014) 757–773.
2. R.J. Gray, A class of K-Sample tests for comparing the cumulative incidence of a competing risk, Ann. Stat. 16 (3) (1988) 1141–1154.
3. Ishwaran H, Kogalur UB, Gorodeski EZ, Minn AJ, Lauer MS (2010). “High-dimensional variable selection for survival data.” J. Amer. Statist. Assoc., 105, 205-217.
4. Ishwaran H, Kogalur UB, Chen X, Minn AJ (2011). “Random Survival Forests for High-Dimensional Data.” Statist. Anal. Data Mining, 4, 115-132.
